# Clinical utility of electrocardiographic voltage parameters for the diagnosis of hypertrophic cardiomyopathy

**DOI:** 10.3389/fcvm.2024.1354364

**Published:** 2024-09-19

**Authors:** Gui Li, Lingdan Jin, Zhiyuan Weng, Xueqing Jin, Xiaoyan Lin, Shuling Chen

**Affiliations:** ^1^ Department of Cardiology, The First Affiliated Hospital of Fujian Medical University, Fuzhou, Fujian, China; ^2^ Department of Ultrasound, The First Affiliated Hospital of Fujian Medical University, Fuzhou, Fujian, China

**Keywords:** electrocardiography, S-L voltage, Peguero voltage, modified Cornell voltage, hypertrophic cardiomyopathy, SD voltage

## Abstract

**Background:**

While electrocardiographic parameters of hypertensive left ventricular hypertrophy (H-LVH) are well known, limited data are available regarding hypertrophic cardiomyopathy (HCM). This study was to assess the diagnostic value of electrocardiographic voltage parameters in HCM.

**Methods:**

Included patients with HCM treated between March 2015 and May 2023. Voltage parameters (S-L, Cornell, Cornell product, Lewis, Peguero, and modified Cornell voltages) and echocardiography were evaluated. Receiver operating characteristic (ROC) curves were used to assess the diagnostic value of electrocardiogram in HCM. The multiple linear regression was conducted to analyze the correlation between electrocardiogram indicators and cardiac diastolic function.

**Results:**

The highest sensitivity for HCM was Peguero voltage (70.4%; 88.6% specificity). The Peguero voltage had high sensitivity in male (63.8%) and female patients (84.9%), those aged <65 years (71.6%) and ≥65 years (68.3%), with non-apical HCM (AHCM) (76.7%), obstructive HCM (82.1%), and non-obstructive HCM (66.9%). The sensitivity of the S-L voltage was high in AHCM (72.2%). The sensitivity for HCM reached 88.7% when the S-L and Peguero voltages were combined. The modified Cornell index had the highest area under the curve (0.88, 95% CI: 0.84–0.91), and its optimal cutoff value was 2.05 mV in males (77.6% sensitivity and 74% specificity) and 1.935 mV in females (90.6% sensitivity and 91.4% specificity). Peguero voltage (beta = 0.154, *P* = 0.034) and S_D_ (beta = 0.223 *P* = 0.004) were independently correlated with E/e’, an index of left ventricular diastolic function.

**Conclusion:**

The Peguero voltage had high sensitivity and specificity for detecting the presence of HCM. It was positively correlated with E/e’ in patients with HCM. For AHCM, the S-L voltage was more advantageous. Combining the S-L voltage with the Peguero voltage further improves the sensitivity for HCM and thus could be used to improve the screening of HCM in clinical practice. The S_D_ and modified Cornell voltage also had good diagnostic performance, especially in females.

## Introduction

Hypertrophic cardiomyopathy (HCM) is one of the major causes of sudden death in adolescents and athletes (1). Early identification of risk factors and effective interventions could prevent adverse outcomes in patients with HCM. Color Doppler ultrasonography is one of the most commonly used screening tools for HCM, increased wall thickness and pressure gradient at the outflow tract detected by ultrasound are crucial for HCM diagnosis and risk stratification, however, HCM subtypes such as apical hypertrophic cardiomyopathy (AHCM) can be easily missed by color Doppler ultrasonography; Although a cardiac magnetic resonance (CMR) can provide more detailed information of heart morphology and is currently regarded as the gold standard for non-invasive heart function evaluation and myocardial fibrosis evaluation, the preparation and price limit its clinical application (2–4). Therefore, the electrocardiogram (ECG) has considered to be a valuable screening tool for HCM, even though some patients with HCM show a normal routine ECG, most HCM patients still present with abnormal ECG findings (5). However, the gold standard for diagnosing HCM remains ultrasonography (6).

The ECG changes are associated with ventricular hypertrophy in QRS amplitude and duration, and these changes have been validated directly or indirectly by autopsy, clinical features, or imaging findings (7). In 2009, 36 ECG criteria were recommended for diagnosing left ventricular hypertrophy (LVH) (8), including the conventional S-L voltage (9), Cornell voltage (10), Cornell product (11), and Lewis voltage (12). These conventional diagnostic parameters have relatively high specificity for detecting the presence of LVH (8) and can predict cardiovascular risks (13, 14). As one of the most common causes of LVH, the ECG pattern of HCM has been frequently used for the evaluation of HCM phenotype and the differential diagnosis of HCM, Vitale et al. compared the ECG pattern of HCM patients and patients with Farby disease and showed that prolonged QRS duration, R_aVL_ ≥1.1 mV and inferior ST depression could independently predict AFD (15, 16). Unfortunately, they have low sensitivity (<50%) and low consistency with left ventricular mass measured by imaging examinations (8, 17). Peguero et al. (18) proposed a novel ECG indicator to detect the presence of LVH, obtained by adding the deepest S wave (S_D_) amplitude in any single lead to the S wave amplitude of lead V4 (S_V4_). S_D _+ S_V4_ has been reported to have high sensitivity, specificity, accuracy, and reproducibility in diagnosing LVH (19, 20). In the general population and patients with cardiovascular disease, ischemic heart disease, and heart failure, Peguero's criteria for diagnosing LVH predicted an increased risk of death, similar to the traditional Cornell voltage or S-L criteria (21). In addition, Peguero's criteria were independently associated with cardiovascular mortality in hemodialysis patients (22). The Peguero voltage (S_D _+ S_V4_) can predict sudden cardiac death and all-cause mortality risk in the general population (21, 23). It is also independently associated with all-cause mortality in patients with aortic valve stenosis (24). Nevertheless, some studies reported that the Peguero voltage has low specificity (24, 25) and is inadequate for predicting cardiovascular death in clinical practice (26). In 2020, Xu et al. (27) proposed a modified Cornell voltage (RAVL + S_D_) by combining Cornell and Peguero voltages. The modified Cornell standard was found to be correlated with the severity of left ventricular configuration changes in patients with essential hypertension. It could also dynamically reflect the hypertensive left ventricular configuration in male patients; its diagnostic value for hypertensive LVH was better than Cornell voltage and S-L voltage (27).

Few studies have examined whether these parameters could be used to screen for HCM. In addition, recent data suggest that ECG screening for HCM should be tailored to ethnicity (28). Therefore, the present study aimed to investigate whether the Peguero voltage and the modified Cornell could be used to screen for HCM in a Chinese population.

## Materials and methods

### Subjects

This retrospective study included patients with HCM and healthy controls admitted to our hospital between March 2015 and May 2023. This study has been carried out in accordance with the Declaration of Helsinki (2000) of the World Medical Association. The study was approved by the Ethics Committee of our hospital. Informed consent was waived by the committee because of the retrospective nature of the study.

For HCM patients, the inclusion criteria was Inpatients with diagnosis of HCM according to the ICD-10 code in the medical history system or outpatients with diagnosis of HCM made by color Doppler ultrasonography (6). The exclusion criteria were: (1) patients with other diseases causing LVH, such as hypertension, aortic stenosis, amyloidosis, Farby disease, Danon Disease, etc.; (2) other cardiomyopathies, such as dilated cardiomyopathy, restrictive cardiomyopathy, arrhythmogenic right ventricular cardiomyopathy, viral cardiomyopathy, or secondary cardiomyopathy; (3) congenital heart disease, valvular heart disease, postinfarction chronic coronary heart disease, or right ventricular hypertrophy; (4) ECG showing complete left and right bundle-branch block, pre-excitation syndrome, ventricular rhythm, or pacing rhythm; (5) patients with diseases that could lower the ECG signal, including obesity, emphysema, hydropericardium, or pneumothorax; or (6) poor-quality ultrasound images or insufficient imaging data.

The healthy controls were selected from individuals who attended a medical examination at the health management center of our hospital during the same period. The inclusion criteria were: Color Doppler echocardiography did not indicate hypertrophic cardiomyopathy, and the left ventricular mass index was <115 g/m^2^ in males and <95 g/m^2^ in females (29). The exclusion criteria were similar to the patients with LVH.

### Data collection and definition

Clinical data including age, sex, height, body weight, and echocardiography data, were collected for all subjects from the medical records. Body mass index (BMI, kg/m^2^) and body surface area (BSA, BSA = [0.006 × height (cm)] + [0.013 × body weight (kg)]−0.153) were calculated.

Echocardiographic measurements including interventricular septum thickness (IVST), left ventricular posterior wall thickness (LVPWT), left ventricular mass (LVM), left atrial diameter (ALD), left ventricular ejection fraction (LVEF), and the peak of early diastolic transmitral inflow velocity/peak of early diastolic mitral annulus velocity (E/e’) were collected, and the LVM index (LVMI) was calculated accordingly (30). Left ventricular outflow tract gradient (LVOTG) was also recorded to divide the HCM patients into obstructive (LVOTG >30 mmHg at rest or during an exercise load) and non-obstructive (LVOTG <30 mmHg at rest and during an exercise load) subgroups.

ECG acquisition and processing was performed using Cardiofax M 1350P,(NIHON KOHDEN, Japan). The components collected in this study included the S wave amplitude of lead V1 and lead V4 (S_V1_ and S_V4_), the R wave amplitude of lead V5 (R_V5_), the R wave amplitude of lead aVL (R_aVL_), and the deepest S wave (S_D_). S-L voltage was defined as S_V1_ + R_V5_, and the threshold value for S-L voltage-based LVH prediction was >3.5 mV (9). The Cornell voltage was calculated as S_V3_ + R_aVL_, Cornell voltage ≥2.8 mV in males and ≥2.0 mV in females suggesting the presence of LVH (10). The modified Cornell index was calculated as S_D_ + R_aVL_, but no threshold value has been previously published for diagnosing LVH. The Cornell product was calculated as (R_AVL _+ S_V3_) × QRS duration for males and (R_AVL _+ S_V3 _+ 0.8) × QRS duration for females, and a threshold value of ≥2.44 mm•s was used for diagnosing LVH (11). A Lewis voltage was defined as (RI–SI) + (SIII–RIII), and the threshold value of ≥1.7 mV was used (12). The Peguero voltage was calculated as S_D _+ S_V4_, and the threshold value for LVH was ≥2.8 mV in males and ≥2.3 mV in females (18).

### Statistical analysis

SPSS 23.0 software (IBM, Armonk, NY, USA) was used for the statistical analysis. Continuous variables are presented as mean ± standard deviation and compared using Student's *t*-test. Categorical data are presented as *n* (%) and compared using the Chi-squared test. The receiver operating characteristic curve (ROC) and area under the curve (AUC) were used to assess the utility of each ECG parameter in detecting the presence of HCM. The *κ* statistic was used to assess the consistency of the diagnoses with the diagnostic criteria for HCM. Multiple linear regression was used to analyze the correlation between various electrocardiogram indicators (Peguero voltage and S_D_) and cardiac diastolic function (E/e’). A two-sided *P* < 0.05 was considered statistically significant.

### Patient and public involvement

Patients or the public WERE NOT involved in the design, conduct, reporting, or dissemination of this research.

## Results

A total of 370 subjects (all Han nationality) were included in the final analysis, 169 in the HCM group (mean age: 59.5 ± 13.7 years, 68.6% male) and 201 in the control group (mean age: 60.5 ± 13.6 years, 65.2% male) (Table 1). All patients’ characteristics were comparable between the two groups. The S-L voltage, Cornell voltage, Cornell product, modified Cornell, Lewis voltage, Peguero voltage, R_V5_, S_V1_, S_V4_, R_aVL_, S_D_, interventricular septum thickness (IVST), left ventricular posterior wall thickness (LVPWT), LVMI, left atrial diameter (LAD), and E/e’ were all significantly higher in the HCM group than in the control group (all *P* < 0.001) (Table 2). However, the LVEF, of HCM patients was lower than participants in the control group (64.14 ± 6.87 vs. 67.63 ± 4.87, *P* < 0.001) (Table 2).

**Table 1:** Clinical data between the HCM and control groups.

Variable	HCM group (*n* = 169)	Control group (*n* = 201)	*P*-value
Age (years), mean ± SD	59.5 ± 13.7	60.5 ± 13.6	0.883
Age range (years)	18–89	22–89	–
Male sex, *n* (%)	116 (68.6)	131 (65.2)	0.481
BMI (kg/m^2^), mean ± SD	23.2 ± 3.2	23.4 ± 2.9	0.183
Diabetes mellitus, *n* (%)	18 (10.7)	26 (12.9)	0.499
Coronary heart disease, *n* (%)	30 (17.8)	40 (19.9)	0.599
Dyslipidemia, *n* (%)	59 (34.9)	83 (41.3)	0.209

HCM, hypertrophic cardiomyopathy; SD, standard deviation; BMI, body mass index.

**Table 2:** Electrocardiographic and color Doppler ultrasound parameters between the HCM and control groups.

Indicator	HCM group (*n* = 169)	Control group (*n* = 201)	*P*
S-L voltage (mV)	4.06 ± 1.91	2.40 ± 0.71	<0.001
Cornell voltage (mV)	2.27 ± 1.27	1.22 ± 0.51	<0.001
Cornell product (mm*s)	2.56 ± 1.47	1.30 ± 0.51	<0.001
Improved Cornell (mV)	2.99 ± 1.36	1.60 ± 0.50	<0.001
Lewis voltage (mV)	0.52 (1.75)	0.14 (0.91)	<0.001
Peguero voltage (mV)	3.38 ± 1.61	1.89 ± 0.72	<0.001
R_V5_ (mV)	2.64 ± 1.51	1.58 ± 0.54	<0.001
S_V1_ (mV)	1.42 ± 0.81	0.82 ± 0.37	<0.001
R_avL_ (mV)	0.67 ± 0.62	0.29 ± 0.23	<0.001
S_D_ (mV)	2.32 ± 1.06	1.32 ± 0.46	<0.001
S_V4_ (mV)	1.06 ± 0.81	0.58 ± 0.37	<0.001
IVST (cm)	1.74 ± 0.60	0.89 ± 0.10	<0.001
LVPWT (cm)	1.02 ± 0.29	0.83 ± 0.22	<0.001
LVMI (g/m^2^)	157.68 ± 61.95	77.29 ± 14.58	<0.001
LAD (cm)	4.28 ± 0.74	3.53 ± 0.47	<0.001
LVEF (%)	64.14 ± 6.87	67.63 ± 4.87	<0.001
E/e’	14.08 ± 6.27	9.62 ± 2.82	<0.001

Data are presented as means ± standard deviations. HCM, hypertrophic cardiomyopathy; IVST, interventricular septum thickness; LVPWT, left ventricular posterior wall thickness; LVMI, left ventricular mass index; LAD: left atrial diameter; LVEF: left ventricular ejection fraction; E/e’, the peak of early diastolic transmitral inflow velocity/peak of early diastolic mitral annulus velocity.

The AUC values for S-L voltage, Cornell voltage, Cornell product, Lewis voltage, modified Cornell, Peguero voltage, and single echocardiographic component were 0.61–0.88 (all *P* < 0.001) (Supplementary Table S1). The sensitivity was the highest for the Peguero voltage (70.4%), followed by the S-L voltage (58.6%). Notably, the specificity of all these parameters was high (88.6%–99.0%) (Supplementary Table S1).

**Figure 1:**
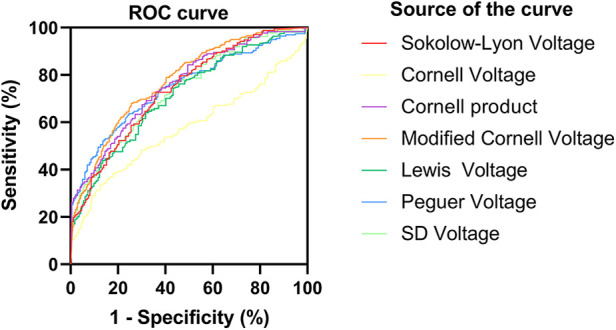
Receiver operating characteristic curve (males). ROC curve; sensitivity; 1-specificity; S-L voltage; Cornell voltage; Cornell product; Lewis voltage; Peguero voltage; S_D_ voltage; Reference line.

**Figure 2:**
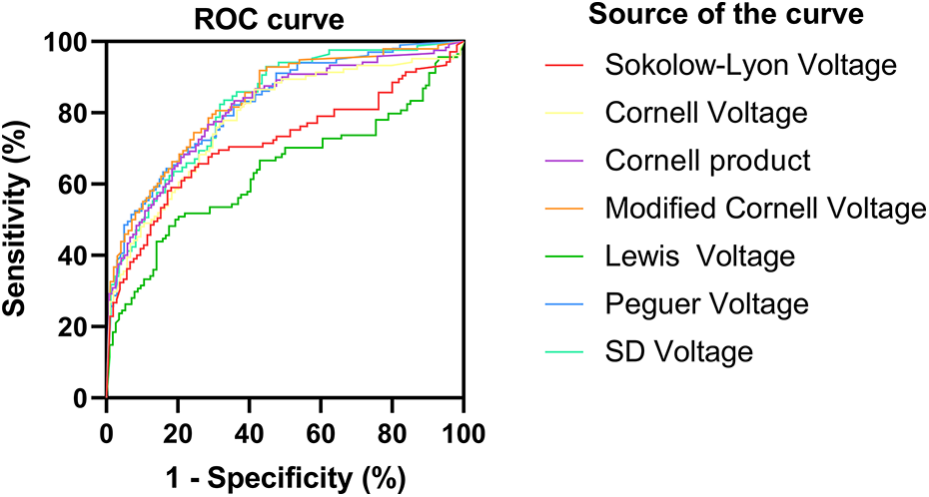
Receiver operating characteristic curve (females). ROC, Receiver operating characteristic curve.ROC curve; sensitivity; 1-specificity; S-L voltage; Cornell voltage; Cornell product; Lewis voltage; Peguero voltage; S_D_ voltage; Reference line.

For HCM detection, the Peguero voltage showed the highest sensitivity and good specificity in both male (AUC, 0.79, 95% CI: 0.74–0.85, sensitivity: 63.8%, specificity: 87%) and female subgroups (AUC, 0.92, 95% CI: 0.87–0.97, sensitivity: 84.9% specificity: 91.4%) (Figures 1, 2 and Supplementary Table S2). The optimal threshold value of the Peguero voltage for detecting the presence of HCM was ≥2.795 mV for males and ≥2.295 mV for females.

The Peguero voltage had the highest sensitivity in those aged <65 years (AUC, 0.85, 95% CI: 0.80–0.90, sensitivity 71.6%) and ≥65 years old (AUC, 0.83, 95% CI: 0.764–0.89, sensitivity 68.3%). The specificity values of the Peguero voltage were relatively high (all >87%) in the younger and older subgroups (Supplementary Table S2).

In the AHCM subgroup, the sensitivity was the highest for the S-L voltage (72.2%) but lower for the Peguero voltage (47.2%) (Supplementary Table S3). In the non-AHCM subgroup, the highest sensitivity was observed for the Peguero voltage (76.7%) (Supplementary Table S3). The Peguero voltage had the highest sensitivity in the obstructive subgroup (82.1%) and the non-obstructive subgroup (66.9%) (Supplementary Table S3).

Although the S-L voltage and Peguero voltage both had relatively high sensitivity for detecting HCM, the S-L voltage showed the best diagnostic performance in AHCM while using them in combination (a diagnosis of HCM was made when any of the S-L voltage or Peguero voltage was positive) resulted in a notable increase in sensitivity, with 88.7% in patients with HCM (86.2% in males and 94.3% in females) (Supplementary Table S4). The specificity of the combined indicator was 81.6% in patients with HCM. The sensitivity of the combined indicator was >80% for all subgroups and was higher than for the S-L voltage or Peguero voltage alone (Supplementary Table S4).

The modified Cornell index had a high AUC in the population (AUC: 0.88, 95% CI: 0.84–0.91) and subgroups (male: AUC: 0.83, 95% CI: 0.78–0.88; female: 0.94, 95% CI: 0.89–0.99; age <65 years: AUC: 0.88, 95% CI: 0.83–0.92; age ≥65 years: 0.88, 95% CI: 0.82–0.93). According to the ROC curve, the optimal cut-off value of the modified Cornell index for the diagnosis of hypertrophic cardiomyopathy was ≥2.05 mV in males (sensitivity and specificity of 77.6% and 74%) and ≥1.935 mV in female patients (sensitivity and specificity of 90.6% and 91.4%). The performance of S_D_ was also evaluated, and the AUC reached 0.94 (95% CI: 0.89–0.99) in females and 0.79 (95% CI: 0.74–0.85) in males, when the cut-off value for males, and females subgroups were set at 1.755 mV and 1.545 mV, the sensitivity was 68% and 89%, while the specificity was 76% and 89% respectively (Supplementary Table S5).

Multiple linear regression analysis shows that after adjusted for sex, age, LVPWT, and Obstructive HCM, Peguero voltage (beta = 0.154, *P* = 0.034, Supplementary Table S6) and S_D_ (beta = 0.223, *P* = 0.004, Supplementary Table S7) were independently positively correlated with E/e’, an index of left ventricular diastolic function.

## Discussion

This study aimed to investigate whether electrocardiographic voltage parameters had value in the diagnosis of HCM. The results revealed that the Peguero voltage had high sensitivity and specificity for detecting the presence of HCM. It was positively correlated with left ventricular diastolic function. For AHCM, the S-L voltage was more advantageous. The modified Cornell voltage also has good diagnostic performance, especially in female patients.

The sensitivity of the combined S-L and Peguero voltages for detecting the presence of HCM was 88.7% in all subjects. In female patients, non-AHCM, the sensitivity was greater than 90%. The sensitivity of this combined indicator was >80% in the other subgroups, and the overall specificity was some 80%. A limitation of the Peguero voltage is its poor sensitivity for AHCM, which could be improved by combining it with the S-L voltage.

In their previous studies, Peguero et al. (18) and Shao et al. (31)mainly focused on patients with hypertension and LVH. A study reported that the diagnostic value of the Peguero voltage was better compared to S-L and Cornell voltages in patients with LVH accompanied by a complete left bundle-branch block (32). In the European population with LVH confirmed by cardiac magnetic resonance (the main causes of LVH included hypertension, hypertrophic cardiomyopathy, dilated cardiomyopathy, valvular heart disease, and invasive cardiomyopathy), the Peguero voltage showed higher sensitivity than the S-L and Cornell voltages (33). Tiron et al. (34) found that in a population of HCM patients, Peguero voltage showed the highest sensibility to identify cardiac hypertrophy compared to S-L voltage and Cornell voltage, and it was the only criterion that correlated with both left ventricular mass index and maximum thickness. Our present study showed that the Peguero voltage had the highest sensitivity in HCM patients (except for AHCM) and it was positively correlated with left ventricular diastolic function. This is consistent with Tiron's research. In AHCM patients, tall R waves and giant T wave inversion in precordial leads are typical presentations, however, not all AHCM patients present with these characteristics and this could partially explain the low sensitivity rate in AHCM patients. The theoretical basis for this observation is that the Peguero voltage measures the highest voltage in all leads instead of the voltage of certain leads and thus could avoid potential bias introduced by the operators, sites of the electrodes, and individual factors such as heart transposition. In addition, measuring the highest voltage in all leads can help identify myocardial hypertrophy at different sites. Second, the new indicator is based on measurements of the S wave instead of the R wave. Theoretically, the latter part of the QRS wave (namely the S wave) might better reflect the vectors caused by the myocardium and free ventricular wall depolarization. The first 30 ms of cardiac depolarization mainly involve depolarization of the interventricular septum, conducting system, and left ventricular endocardium, while the latter 50 ms involve depolarization of the myocardium of the left ventricle and left ventricular epicardium. Therefore, patients with mild or moderate LVH exhibit ECG voltage changes corresponding to the latter part of the QRS wave, namely the S wave. These findings stress the importance of changes in the S wave and those of the R wave. We also showed that the S-L parameter alone could not detect the presence of HCM with a high sensitivity, which is in alignment with a previous study performed by Bayram et, who found sensitivity and accuracy were only 1.9% and 64.9% (35), however, after combination with S-L voltage, the sensitivity could be even higher compared to Puguero along, the S-L voltage was calculated as S_V1_ + R_V5_, according to the description of Sokolow (9), the hypertrophy of left ventricle could induce a stronger electrical signal and lead to a high R_V5_ or R_V6_, the increase on wall thickness and the right directed electrical axis also showed a deep S wave in lead V1. Therefore, the S-L combined with Puguero provides the anatomical and electrical characteristics evaluation simultaneously in the heart of HCM patients.

Most previous studies demonstrated that the sensitivity of the conventional parameters was low for diagnosing LVH. For example, Lv et al. (36) reported that in a general Chinese population, the sensitivity of the S-L voltage and the Cornell voltage was only 12%, with the Cornell product index performing worse, only 4%. Tsiachris et al. (37)reported that the sensitivity of all the conventional parameters was <24% in middle-aged and elderly subjects. This study showed that the modified Cornell index had superior diagnostic performance compared to the traditional Cornell index. The difference between the two indexes was that the modified Cornell index uses S_D_ instead of S_V3_, effectively eliminating the measurement deviation caused by improper operation, position deviation, cardiac transposition, and other reasons, thus improving sensitivity. In this study, the modified Cornell index had a higher AUC in all subgroups, especially in women (when the optimal cutoff value of ≥1.935 mV was used), and its diagnostic sensitivity was higher than that of the Peguero voltage on the premise of the same specificity. The modified Cornell index combines the voltage changes of the frontal and transverse surfaces, R and S waves, and can more comprehensively track the site of ventricular hypertrophy. Moreover, a study reported that R_aVL_ had a stronger correlation with LVH in all single-lead component analyses (36), and therefore, it might account for the good diagnostic performance of the modified Cornell metrics.

In their study, Campbell et al. (38) reported a proprietary algorithm based on voltage and ECG-based Seattle criteria that had 90% sensitivity and 96% specificity for automated ECG screening of HCM. Unfortunately, the exact variables considered in their algorithm are not available. Rahman et al. (39) extracted 304 ECG features based on the standard 10-s, 12-lead ECG that could identify HCM with 90% sensitivity and specificity. Some other scholars developed a new diagnostic standard for LVH by using common electrocardiogram indicators to build an optimized model or establish a scoring system. Although it could improve diagnostic sensitivity compared with traditional indicators, it was not suitable for primary clinical screening due to its complexity (40, 41). Therefore, the value of complex algorithms such as those described above should be tested against more simple parameters to determine if they are worth the increased costs.

A major limitation of the present study was that the participants were mainly aged (mean age of 60.0 ± 14.2 years in the HCM group and 60.5 ± 13.6 years in the control group), and only a few young adults were included. HCM is often misperceived as a disease of the young with poor outcomes, but the age-dependent expression of HCM mutations is well recognized (42). A recent population-based study from Germany reported that the prevalence of HCM dramatically increased from 12.5/100,000 persons in those aged 20–29 years to 149.9/100,000 persons in those aged 60–69 years and 254.9/100,000 persons in those aged 70–79 years. and the average age of the patients diagnosed with HCM was 63 ± 17 years (43), which is comparable to the present study. Finally, it should be noted that HCM is often well tolerated and is usually diagnosed only later in life when initial symptoms are first present (44). Interestingly, previous research suggested that HCM in Chinese people might be associated with a late-onset presentation (45). Considering the above factors, the age distribution of the patients with HCM in this study is not unexpected. Nevertheless, additional studies are needed to evaluate the utility of ECG parameters, including the combination of the S-L voltage and Peguero voltage, in adolescents and young adults. There are some other limitations in the present study. All participants were enrolled from a single centre in China, so the generalizability of the findings is unknown. The diagnosis of HCM was based solely on echocardiography, the CMR was not performed for all participants due to the clinical availability. Genetic analyses were not performed to support the diagnosis of HCM. The only ECG parameters we investigated were those established for the screening of LVH, while other parameters or more complex algorithms were not evaluated. Using an 18-lead ECG or vectorcardiogram would have allowed for a more comprehensive assessment of the ECG parameters. Finally, some subgroups, such as AHCM and obstructive HCM, had small sample sizes.

## Conclusion

In Conclusion, the Peguero voltage has high sensitivity and specificity for detecting the presence of HCM in adults in China. It is positively correlated with the diastolic function of the heart in patients with HCM. For AHCM, the S-L voltage was more advantageous. Combining the S-L voltage with the Peguero voltage further improves the sensitivity for HCM and thus could be used to improve the screening of HCM in clinical practice. The S_D_ and modified Cornell voltage were optional indices for diagnosing HCM, especially in females.

## Data Availability

The original contributions presented in the study are included in the article/Supplementary Material, further inquiries can be directed to the corresponding author.
