# Supplementary material for: Clinical utility of electrocardiographic voltage parameters for the diagnosis of hypertrophic cardiomyopathy
Source: Front Cardiovasc Med. 2024 Sep 19;11:1354364. doi: 10.3389/fcvm.2024.1354364 (PMC11462641; doi:10.3389/fcvm.2024.1354364)
Supplement: Supplementary file 1 [file Table1.docx]

Supplementary Materials

**Table S1.** The ability of the electrocardiographic indicators to detect the presence of hypertrophic cardiomyopathy (n=370)

| ECG indicator | AUC (95%CI) | P | Sensitivity,  n (%) | Specificity,  n (%) | κ |
| --- | --- | --- | --- | --- | --- |
| S-L voltage  Cornell voltage  Cornell product  Lewis voltage  Peguero voltage  Modified Cornell  R_V5_  S_V1_  R_avL_  S_D_  S_V4_ | 0.79 (0.74-0.84)  0.81 (0.77-0.86)  0.83 (0.79-0.88)  0.61 (0.55-0.67)  0.84 (0.80-0.88)  0.88 (0.84-0.91)  0.73(0.67-0.79)  0.75(0.69-0.80)  0.69(0.63-0.74)  0.85(0.81-0.89)  0.71(0.65-0.76) | <0.001  <0.001  <0.001  <0.001  <0.001  <0.001  <0.001  <0.001  <0.001  <0.001  <0.001 | 99 (58.6)  54 (32.0)  68 (40.2)  35 (20.7)  119 (70.4)  -  -  -  -  -  - | 184 (91.5)  197 (98.0)  198 (98.5)  199 (99.0)  178 (88.6)  -  -  -  -  -  - | 0.514  0.316  0.406  0.210  0.597  -  -  -  -  -  - |

AUC, area under the receiver operating characteristic curve; CI, confidence interval; ECG, electrocardiogram; S-L voltage, Sokolow-Lyon voltage. S_D_ voltage, the deepest S wave in 12 lead ECG

**Table S2.** The ability of the electrocardiographic indicators to detect the presence of hypertrophic cardiomyopathy in subgroups based on sex and age

| ECG indicator | AUC (95%CI) | P | Sensitivity, n (%) | Specificity, n (%) | κ |
| --- | --- | --- | --- | --- | --- |
| Males (n=247)  S-L voltage  Cornell voltage  Cornell product  Lewis voltage  Peguero voltage  Modified Cornell  R_V5_  S_V1_  R_avL_  S_D_  S_V4_  Females (n=123)  S-L voltage  Cornell voltage  Cornell product  Lewis voltage  Peguero voltage  Modified Cornell  R_V5_  S_V1_  R_avL_  S_D_  S_V4_  <65 years (n=229)  S-L voltage  Cornell voltage  Cornell product  Lewis voltage  Peguero voltage  Modified Cornell  R_V5_  S_V1_  R_avL_  S_D_  S_V4_  >65 years (n=141)  S-L voltage  Cornell voltage  Cornell product  Lewis voltage  Peguero voltage  Modified Cornell  R_V5_  S_V1_  R_avL_  S_D_  S_V4_ | 0.80(0.75-0.86)  0.77 (0.71-0.83)  0.82 (0.77-0.87)  0.59 (0.52-0.67)  0.79 (0.74-0.85)  0.83 (0.78-0.88)  0.76(0.69-0.82)  0.72(0.66-0.79)  0.66(0.59-0.73)  0.79(0.74-0.85)  0.68(0.61-0.75)  0.77 (0.68-0.86)  0.87 (0.80-0.94)  0.88 (0.82-0.95)  0.65(0.54-0.75)  0.92 (0.87-0.97)  0.94 (0.89-0.99)  0.67(0.56-0.78)  0.80(0.72-0.89)  0.74(0.64-0.83)  0.94(0.90-0.99)  0.75(0.66-0.84)  0.77 (0.71-0.84)  0.82 (0.76-0.87)  0.83 (0.78-0.88)  0.63 (0.56-0.71)  0.85 (0.80-0.90)  0.88 (0.83-0.92)  0.69(0.61-0.76)  0.77(0.71-0.84)  0.73(0.67-0.80)  0.84(0.79-0.89)  0.72(0.65-0.79)  0.83 (0.75-0.90)  0.80 (0.73-0.88)  0.84 (0.77-0.91)  0.58 (0.48-0.69)  0.83 (0.76-0.89)  0.88 (0.82-0.93)  0.81(0.73-0.90)  0.70(0.60-0.79)  0.62(0.52-0.72)  0.86(0.80-0.92)  0.69(0.60-0.78) | <0.001  <0.001  <0.001  0.011  <0.001  <0.001  <0.001  <0.001  <0.001  <0.001  <0.001  <0.001  <0.001  <0.001  0.005  <0.001  <0.001  <0.001  <0.001  <0.001  <0.001  <0.001  <0.001  <0.001  <0.001  <0.001  <0.001  <0.001  <0.001  <0.001  <0.001  <0.001  <0.001  <0.001  <0.001  <0.001  0.087  <0.001  <0.001  <0.001  <0.001  <0.001  <0.001  <0.001 | 67 (57.8)  21 (18.1)  36 (31.0)  22 (19.0)  74 (63.8)  -  -  -  -  -  -  32 (60.4)  33 (62.3)  32 (60.4)  13 (24.5)  45 (84.9)  -  -  -  -  -  -  62 (56.9)  37 (33.9)  47(43.1)  22 (20.2)  78 (71.6)  -  -  -  -  -  -  37(61.7)  17 (28.3)  21(35.0)  13 (21.7)  41 (68.3)  -  -  -  -  -  - | 119 (90.8)  131 (100)  129 (98.5)  130 (99.2)  114 (87.0)  -  -  -  -  -  -  65 (92.9)  66 (94.3)  69 (98.6)  69 (98.6)  64 (91.4)  -  -  -  -  -  -  112 (93.3)  118 (98.3)  117 (97.5)  118 (98.3)  107 (89.2)  -  -  -  -  -  -  72 (88.9)  79 (97.5)  81 (100)  81 (100)  71 (87.7)  -  -  -  -  -  - | 0.495  0.190  0.307  0.191  0.515  -  -  -  -  -  -  0.553  0.587  0.618  0.254  0.767  -  -  -  -  -  -  0.511  0.333  0.417  0.192  0.612  -  -  -  -  -  -  0.521  0.284  0.382  0.241  0.571  -  -  -  -  -  - |

AUC, area under the receiver operating characteristic curve; CI, confidence interval; ECG, electrocardiogram; S-L voltage, Sokolow-Lyon voltage

**Table S3.** Comparisons of the sensitivities of the electrocardiographic indicators for detecting the presence of hypertrophic cardiomyopathy between subgroups based on different types of hypertrophic cardiomyopathy

| Subgroup | S-L voltage | Cornell voltage | Cornell product | Lewis voltage | Peguero voltage |
| --- | --- | --- | --- | --- | --- |
| AHCM (n=36)  Non-AHCM (n=133)  P  OHCM (n=39)  Non-OHCM (n=130)  P | 72.2%  54.9%  0.061  56.4%  59.2%  0.754 | 11.1%  37.6%  0.003  48.7%  26.9%  0.010 | 22.2%  45.1%  0.013  59.0%  34.6%  0.007 | 5.6%  24.8%  0.011  35.9%  16.2%  0.008 | 47.2%  76.7%  0.001  82.1%  66.9%  0.069 |

AHCM, apical hypertrophic cardiomyopathy; OHCM, Obstructive hypertrophic cardiomyopathy; HT, hypertension; S-L voltage, Sokolow-Lyon voltage

**Table S4.** Comparisons of the abilities of the Sokolow-Lyon voltage, Peguero voltage, and their combination to detect the presence of HCM

| Subgroup | S-L voltage | | Peguero voltage | | | S-L+Peguero voltages | | |
| --- | --- | --- | --- | --- | --- | --- | --- | --- |
|  | Sensitivity | Specificity | | Sensitivity | Specificity | | Sensitivity | Specificity |
| All subjects  Males  Females  <65 years old  ≥65 years old  AHCM  Non-AHCM  OHCM  Non-OHCM | 58.6%  57.8%  60.4%  56.9%  61.7%  72.2%  54.9%  56.4%  59.2% | 91.5%  90.8%  92.9%  93.3%  88.9%  -  -  -  - | | 70.4%  63.8%  84.9%  71.6%  68.3%  47.2%  76.7%  82.1%  66.9% | 88.6%  87.0%  91.4%  89.2%  87.7%  -  -  -  - | | 88.7%  86.2%  94.3%  88.1%  90.0%  80.6%  91.0%  89.7%  88.5% | 81.6%  79.4%  85.7%  84.2%  77.8%  -  -  -  - |

AHCM, apical hypertrophic cardiomyopathy; OHCM, Obstructive hypertrophic cardiomyopathy; HT, hypertension; S-L voltage, Sokolow-Lyon voltage

**Table S5.** Best cut-off value of modified Cornell and S_D_ in HCM patients

| ECG indicator | AUC (95CI%) | cut-off value (mv) | Sensitivity | Specificity |
| --- | --- | --- | --- | --- |
| Modified Cornell  (males) | 0.83(0.78-0.88) | 2.05 | 77.6% | 74.0% |
| Modified Cornell  (females)  S_D_  (males)  S_D_  (females) | 0.94(0.89-0.99)  0.79(0.74-0.85)  0.94(0.90-0.99) | 1.935  1.755  1.545 | 90.6%  68%  89% | 91.4%  76%  89% |

**Table S6.** Correlation between Peguero voltage and E/e’ by multivariate linear regression

| Influencing factor | Standardized coefficient Beta | P |
| --- | --- | --- |
| Peguero voltage  Sex | 0.154  -0.151 | 0.034*  0.023* |
| Age | 0.203 | 0.003* |
| LVPWT  Obstructive HCM | 0.395  0.271 | <0.001**  <0.001** |

*, P<0.05; **, P<0.001

**Table S7.** Correlation between SD E/e’ by multivariate linear regression

| Influencing factor | Standardized coefficient Beta | P |
| --- | --- | --- |
| S_D_  Sex | 0.223  -0.148 | 0.004*  0.023* |
| Age | 0.221 | 0.001* |
| LVPWT  Obstructive HCM | 0.342  0.271 | <0.001**  <0.001** |

*, P<0.05; **, P<0.001
